# Supplementary material for: Small Molecule 20S Proteasome Enhancer Regulates MYC Protein Stability and Exhibits Antitumor Activity in Multiple Myeloma
Source: Biomedicines. 2022 Apr 19;10(5):938. doi: 10.3390/biomedicines10050938 (PMC9138505; doi:10.3390/biomedicines10050938)
Supplement: Supplementary file 1 [file biomedicines-10-00938-s001.zip › biomedicines-1654325-supplementary r1.pdf]

List of Supplementary Materials:

Figure S1: Induction of caspase 3/7 activity using Caspase-Glo 3/7 Assay

Figure S2: Cell viability curves for Table 1 data

Figure S3: Pharmacokinetic data in mice.

Figure S4: Weights of mice during the xenograft study.

Figure S5: Tumor volume data from TCH-165 treated RPMI-8226 xenograft model using SCID mice.

Figure S6: Pharmacokinetic data in dogs.

Figure S7: Target engagement study in treated and untreated dogs.

Table S1: Blood count panel of treated versus untreated dogs.

Table S2: Clinical chemistry panel of treated versus untreated dogs.

Table S3: Normalized gene expression data for the RPMI-8226 cell line using the Nanostring PanCancer Pathways Panel (see companion excel file for the table).

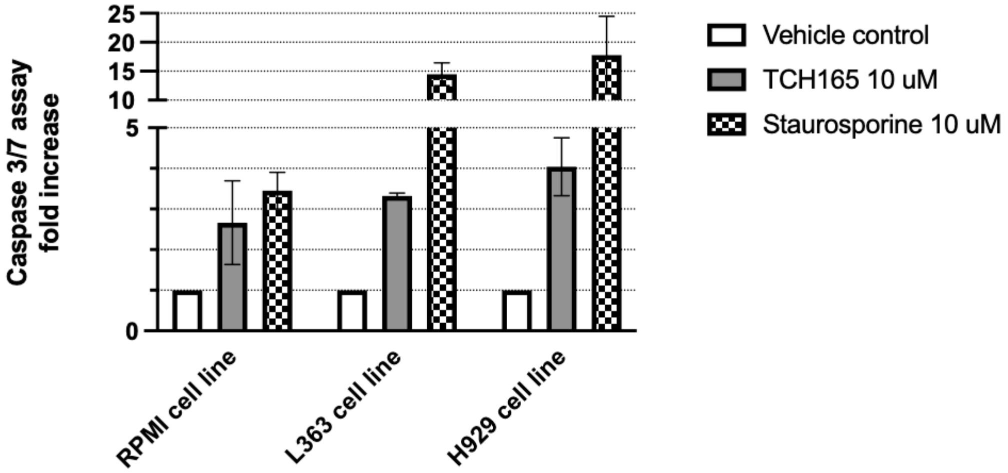

Figure S1. Induction of caspase 3/7 activity using Caspase-Glo 3/7 Assay. RPMI-8226, L363 and H929 cells were treated with vehicle (control), TCH-165 (10 mM), and Staurosporine (10 mM, positive control) for 24 hours (n=3).

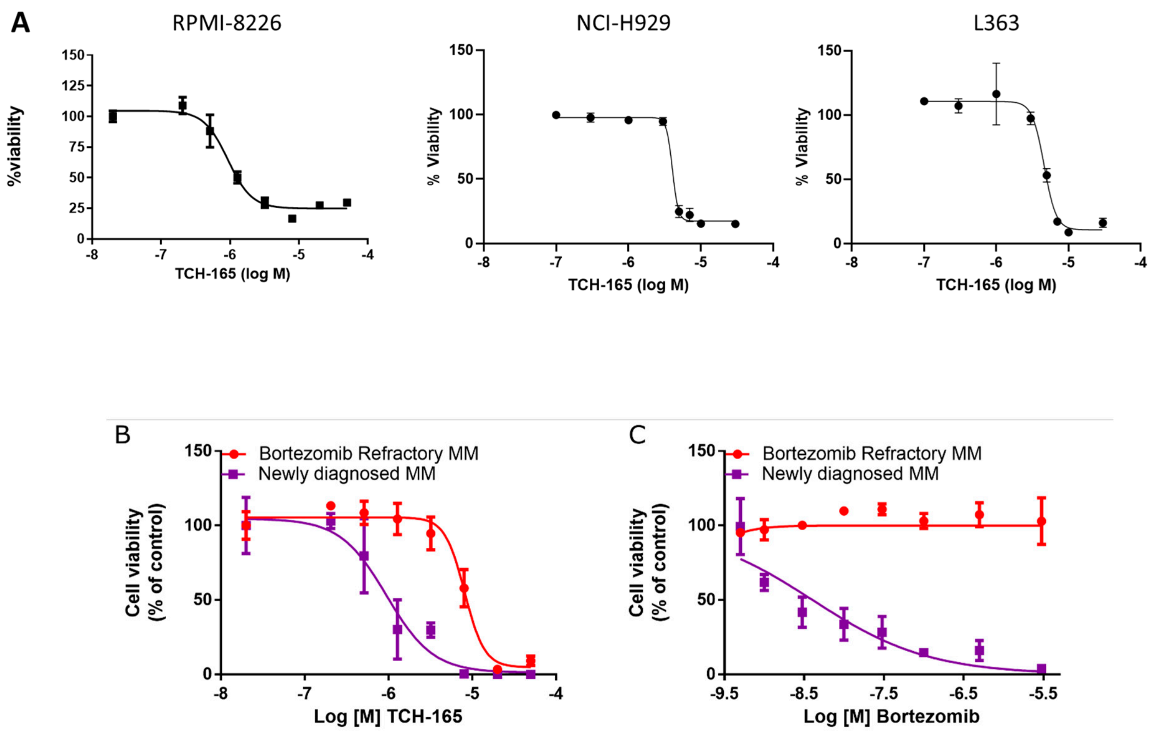

Figure S2: Cell viability curves for Table 1 data. **(A)** multiple myeloma cells, RPMI-8226, NCI-H929 and L363 multiple myeloma cells following treatment with TCH-165 for 72h. **(B + C)** Multiple myeloma cells isolated from bone marrow aspirates of a newly diagnosed patient or a patient who is inherently resistant to bortezomib treatment were treated with TCH-165 (**B**) or Bortezomib (**C**) and cell viability measured after 72h.

Group	PK Timepoint (h) Post-Initial Gavage	Animal #	Plasma Concentration (nM)	Mean Plasma Concentration (nM)	± Standard Deviation
1	0.5	1	447.6	355.1	181.5
		2	146.0		
		3	471.6		
2	1	4	198.0	620.8	398.6
		5	989.6		
		6	674.8		
3	2	7	819.2	932.3	184.5
		8	832.4		
		9	1145.2		
4	4	10	764.0	678.9	323.3
		11	951.2		
		12	321.6		
5	8	22	885.6	845.9	373.6
		23	1198.0		
		24	454.0		
6	9	16	1321.2	1170.9	130.6
		17	1107.2		
		18	1084.4		
7	10	19	646.0	911.6	282.7
		20	880.0		
		21	1208.8		
8	12	13	2138.4	1434.0	625.2
		14	1218.8		
		15	944.8		
9	16	25	840.4	471.1	330.5
		26	203.2		
		27	369.6		

Figure S3. Pharmacokinetic data in mice. TCH-165 plasma concentrations after oral gavage 3:7 (v/v) propylene glycol: 5% D5W vehicle of male CD-1 mice.

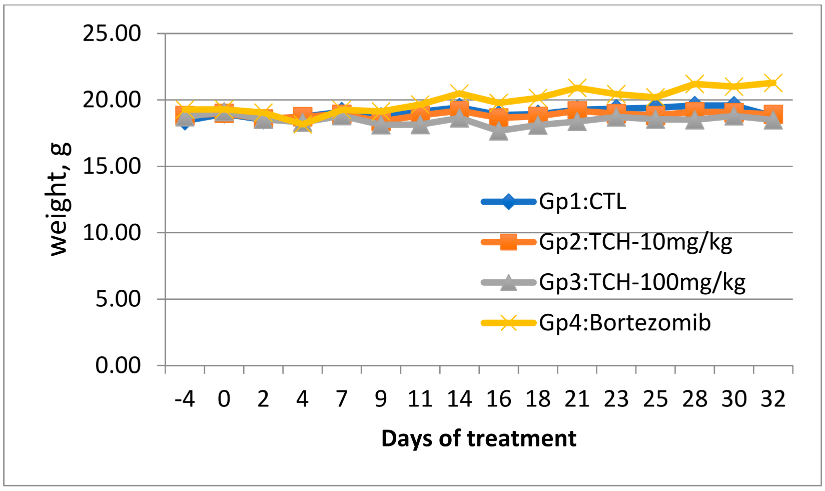

Figure S4. Weights of treatment groups of study (see Figure 5B) in RPMI-8226 subcutaneous xenograft model in SCID mice treated with vehicle control (3:7 (v/v) propyl-ene glycol/5% dextrose), and TCH-165 (10 mg/Kg and 100 mg/kg, bid with oral gavage), bortezomib was at 0.375 mg/kg-day 0, 0.18 mg/kg-day 2, 0.09 mg/kg day 5 and beyond) was given 3X per week intravenously.

Mean	Days	-17	-14	-11	-7	-3	0	2	4	7	9	11
Gp1: Control		10.60	15.43	43.95	41.76	38.49	63.25	70.62	90.73	137.86	163.48	212.01
Gp2:TCH-10mg/kg		9.68	16.32	30.88	45.85	36.36	58.33	55.23	70.72	89.68	98.72	103.98
Gp3:TCH-100mg/kg		11.18	19.37	32.31	37.50	38.99	49.70	47.20	54.74	73.95	74.03	79.59
Gp4:Bortezomib		10.30	8.87	18.10	28.61	37.38	47.25	69.28	52.54	62.66	64.45	104.43

14	16	18	21	23	25	28	30	32	35	37	39	42
241.02	319.30	346.02	466.65	478.21	561.67	786.61	841.01	900.52	958.86	1026.87	1122.13	1253.46
138.48	148.48	230.71	281.51	343.57	395.40	569.20	739.04	733.71	867.08	1028.36	1046.77	1395.61
84.60	96.08	112.96	107.12	142.69	145.59	169.57	177.03	198.99	239.73	252.08	270.05	304.43
122.46	126.51	137.34	158.57	204.03	264.35	287.39	323.49	363.80	396.01	510.98	617.56	771.41

Figure S5. Tumor volume data from TCH-165 treated RPMI-8226 xenograft model using SCID mice.

A

	Dog 1	Dog 2	Dog 3	Mean	SD	N
**Pre-dose**	0	0	0	0	0	3
**1hr SD1**	25	0	23	16	14	3
**2hr SD1**	103	63	160	109	49	3
**4hr SD1**	183	121	97	134	44	3
**8hr SD1**	130	41	45	72	50	3
**9hr SD1**	534	453	33	340	269	3
**10hr SD1**	1444	1390	239	1024	681	3
**24hr SD1**	909	2199	1603	1570	645	3
**1hr SD5**	593	961	750	768	184	3
**2hr SD5**	605	881	792	759	141	3
**4hr SD5**	747	1017	595	786	214	3
**8hr SD5**	756	782	387	641	221	3
**9hr SD5**	841	873	350	688	293	3
**10hr SD5**	1622	1462	733	1272	474	3
**24hr SD5**	546	1225	1135	969	369	3

B

	Dog 1	Dog 2	Dog 3	Mean
**$AUC_{(0-24)}$ Day 1 (nM)**	18781	2631	13713	19772
**Cmax Day 1 (nM)**	1444	2199	1603	1570
**Tmax Day 1 (nM)**	10	24	24	24
**$AUC_{(0-24)}$ Day 5 (nM)**	22163	27221	18108	22494
**Cmax Day 5 (nM)**	1622	1462	1135	1272

Figure S6. Pharmacokinetic data in dogs. (**A**) Plasma concentration (nM) of TCH-165 following oral gavage (500mg BID) at Day 1 (SD1) and Day 5 (SD5). (**B**) Pharmacokinetic parameter of TCH-165 (500mg BID).

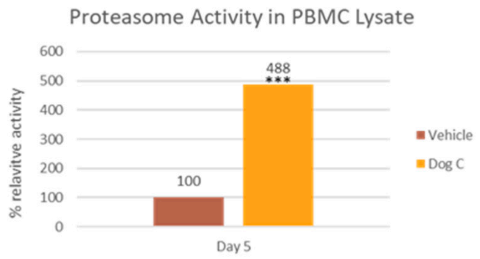

Dog 1					
Trial 1		Trial 2		Trial 3	
Vehicle	Day 5	Vehicle	Day 5	Vehicle	Day 5
0.019	0.081	0.017	0.087	0.016	0.085
0.018	0.079	0.015	0.083	0.020	0.089
0.017	0.077	0.015	0.086	0.017	0.084
**0.018**	**0.079**	**0.016**	**0.085**	**0.018**	**0.086**
4.39		5.45		4.87	

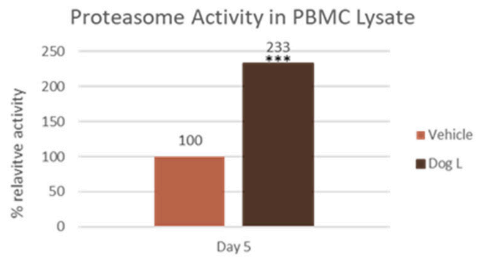

Dog 2					
Trial 1		Trial 2		Trial 3	
Vehicle	Day 5	Vehicle	Day 5	Vehicle	Day 5
0.038	0.082	0.041	0.093	0.044	0.107
0.036	0.083	0.032	0.090	0.042	0.101
0.036	0.077	0.044	0.093	0.042	0.089
**0.037**	**0.081**	**0.039**	**0.092**	**0.043**	**0.099**
2.20		2.36		2.32	

Figure S7. Target engagement study in treated and untreated dogs.

		Pre Dose			Day 6		
		Dog 001	Dog 002	Dog 003	Dog 001	Dog 002	Dog 003
**Hemolysis**		Normal	Normal	Normal	Normal	Normal	Normal
**Lipemia**		Normal	Normal	Normal	Normal	Normal	Normal
**Icterus**		Normal	Normal	Normal	Normal	Normal	Normal
**Total Protein**	*g/dL*	6.7	6.4	6.8	7	6.4	6.4
**RBC**	*x 10.e6/uL*	7.1	6.8	7	7.3	7.4	6.2
**Hgb**	*g/dL*	16.1	15.9	16.3	16.8	17.6	14.9
**Hct**	*%*	47	46	47	50	50	44
**HCT Spun**	*%*	48	44	45	50	51	45
**MCV**	*fL*	66	67	68	68	69	70
**MCH**	*pg*	23	23	23	23	24	24
**MCHC**	*g/dL*	34.0	35.0	34.0	34.0	35.0	34.0
**CHCM**	*g/dL*	33*	34	34	32*	33	32*
**RDW**	*%*	13**	13**	13**	13**	12	12
**Platelet**	*x10.e3/uL*	287	269	188	269	242	151*
**MPV**	*fL*	10.4	9.4	13.7	11	10.4	15.1 **
**WBC**	*x10.e3/uL*	9.7a	10.9	8.2	9.4	10.8	7.9
**Seg Neut #**	*x10.e3/uL*	5.7	7.1	5.7	NA	NA	NA
**Neutrophil #**	*x10.e3/uL*	NA	NA	NA	5.9	6.1	4.5
**Band Neutrophil #**	*x10.e3/uL*	0.1	0.2**	0.1	NA	NA	NA
**Lymphocyte #**	*x10.e3/uL*	2.8	3.1	1.6	2.6	3.8**	2.5
**Monocyte #**	*x10.e3/uL*	0.6	0.3	0.8	0.6	0.5	0.6
**Eosinophil #**	*x10.e3/uL*	0.5	0.2	0	0.3	0.3	0.2
**Basophil #**	*x10.e3/uL*	0	0	0	NA	NA	NA
**LUC #**	*x10.e3/uL*	NA	NA	NA	NA	NA	0.03
**Neutrophil Pct**	*%*	NA	NA	NA	62.9	56.7	56.8
**Seg Neut Pct**	*%*	59	65	70	NA	NA	NA
**Band Neut Pct**	*%*	1	2	1	NA	NA	NA
**Lymphocyte Pct**	*%*	29	28	19	27.1	35.2	31.3
**Monocyte Pct**	%	6	3	10	6.1	4.8	7.8
**Eosinophil Pct**	%	5	2	0	3	2.8	3.2
**Basophil Pct**	%	0	0	0	NA	NA	NA
**LUC Pct**	%	1	NA	NA	0.4	0.2	0.3
**NRBC**	/100 WBC	1	NA	NA	NA	NA	NA
**NRBC #**	*x10.e3/uL*	0.1	NA	NA	NA	NA	NA
**Reactive Lymphs**		NA	NA	NA	Present	Present	NA
**Platelet Clump**		NA	NA	present	Present	Present	Present
**Platelet Comment**		NA	NA	b	b	b	b

*** Low Result**

**** High Result**

a - WBC corrected for nucleated RBCs

b - Platelet concentration should be considered a minimum value and the MPV may be falsely increased due to platelet clumping.

Table S1. Blood count panel of treated versus untreated dogs.

		Pre-Dose			24h Post Day 5 AM Dose		
		Dog 001	Dog 002	Dog 003	Dog 001	Dog 002	Dog 003
Urea Nitrogen	mg/dL	20	16	21	17	14	18
Creatinine	mg/dL	1	0.8	0.9	0.8	0.6	0.7
Sodium	mmol/L	148	148	147	146	144	146
Potassium	mmol/L	4.5	4.4	4.7	4.7	4.6	4.4
Chloride	mmol/L	111	111	106	110	112	110
TCO2	mmol/L	23	24	27	22	18	22
Anion Gap	mmol/L	18	17	19	19	19	18
Na/K Ratio		33	34	31	31	31	33
Osmolarity Calc	mOs/L	308	307	306	303	298	303
Glucose	mg/dL	88	92	87	91	83	87
Calcium	mg/dL	10.1	10.0	10.2	10.3	10.3	9.9
Magnesium	mg/dL	1.9	1.9	2	1.8	1.6*	1.7
Phosphorus	mg/dL	4.4	4.6	4.9	4.3	4.4	4.3
Iron	ug/dL	126	147	108*	276**	284**	307**
Total Protein	g/dL	5.9	5.7	5.9	5.4	5.0*	5.0*
Albumin	g/dL	3.3	3.2	3.2	3.1	3	2.8
Globulin Calc	g/dL	2.6	2.5	2.7	2.3	2.0*	2.2*
Total Bili	mg/dL	0.2	0.2	0.2	0.1	0.2	0.1
Direct Bili	mg/dL	0	0	0	0	0	0
Indirect Bili	mg/dL	0.2	0.2	0.2	0.1	0.2	0.1
Amylase	U/L	612	457	590	495	505	516
ALP	U/L	59	32	35	66	71	47
ALT	U/L	19*	34	24	25	44	23
AST	U/L	26	37	34	23	32	24
Chol	mg/dL	111	330**	125	184	140	190
CK	U/L	180	136	203	78	241**	83
Hemolysis		Normal	Normal	Normal	Normal	Slight	Normal
Icterus		Normal	Normal	Normal	Normal	Normal	Normal
Lipemia		Normal	Normal	Normal	Normal	Normal	Normal

* Low Result

** High Result

Table S2. Clinical chemistry panel of treated versus untreated dogs.

Table S3. See associated excel file for full table.
